# Epstein–Barr virus BRLF1 induces genomic instability and progressive malignancy in nasopharyngeal carcinoma cells

**DOI:** 10.18632/oncotarget.20695

**Published:** 2017-09-05

**Authors:** Sheng-Yen Huang, Chung-Chun Wu, Yu-Jhen Cheng, Sheng-Ping Chou, Yun-Jin Jiang, Kuo-Chang Chu, Ching-Hwa Tsai, Su-Fang Lin, Jen-Yang Chen

**Affiliations:** ^1^ National Institute of Cancer Research, National Health Research Institutes, Miaoli 35053, Taiwan; ^2^ Institute of Molecular and Genomic Medicine, National Health Research Institutes, Miaoli 35053, Taiwan; ^3^ Department of Microbiology, College of Medicine, National Taiwan University, Taipei 10051, Taiwan

**Keywords:** BRLF1, Epstein–Barr virus, nasopharyngeal carcinoma, chromosome mis-segregation, genomic instability

## Abstract

Nasopharyngeal carcinoma (NPC) is a serious health problem in China and Southeast Asia. Relapse is the major cause of mortality, but mechanisms of relapse are mysterious. Epstein–Barr virus (EBV) reactivation and host genomic instability (GI) have correlated with NPC development. Previously, we reported that lytic early genes DNase and BALF3 induce genetic alterations and progressive malignancy in NPC cells, implying lytic proteins may be required for NPC relapse. In this study, we show that immediate early gene BRLF1 induces chromosome mis-segregation and genomic instability in the NPC cells. Similar phenomenon was also demonstrated in 293 and zebrafish embryonic cells. BRLF1 nuclear localization signal (NLS) mutant still induced genomic instability and inhibitor experiments revealed that BRLF1 interferes with chromosome segregation and induces genomic instability by activating Erk signaling. Furthermore, the chromosome aberrations and tumorigenic features of NPC cells were significantly increased with the rounds of BRLF1 expression, and these cells developed into larger tumor nodules in mice. Therefore, BRLF1 may be the important factor contributing to NPC relapse and targeting BRLF1 may benefit patients.

## INTRODUCTION

Nasopharyngeal carcinoma (NPC) is a squamous-cell carcinoma derived from the nasopharyngeal epithelium of the post nasal cavity. It is rare worldwide but has a very unique pattern of distinct ethnic and geographic distribution such as southern China, Southeast Asia, northeast India and North Africa [1, 2]. NPC is radiosensitive and the primary mode of treatment is radiotherapy. However, chemoradiotherapy has been shown to be better than radiotherapy alone in patients with advanced NPC [3]. Recently, a significant increase in the survival rate has been achieved with improvements of combinatory radiotherapy and neoadjuvant chemotherapy. If treatment is started at early stages, the 5-year survival rate may be as high as 80-95%. However, if the treatment is started at late stages, the 5-year survival rate is poor [4]. Although high risk patients can be treated with remission, local relapse and distant metastasis become the major causes of mortality. Therefore, prevention of relapse and metastasis appears to be the most important issue in the control of NPC. To cope with this clinical difficulty, delineation of the mechanism(s) of NPC relapse and metastasis is imperative.

In the carcinogenesis of NPC, genetic factors, consumption of nitroso-compounds and Epstein–Barr virus (EBV) infection have been found to play important roles [5, 6]. EBV is a member of the *herpesviridae*. The life cycle includes latent and lytic stages. The shift from latency to the lytic cycle is known as reactivation [7] and is initiated by two immediate early viral proteins, BRLF1 and BZLF1. Upon reactivation, there is a cascade of expression of lytic genes: the immediate early (IE) genes BZLF1 and BRLF1 transactivate the early (E) genes, including DNase and BALF3, followed by the expression of late (L) genes, including VCA [8]. Elevation of antibodies against EBV was first detected in patients with NPC [9]. Elevation of antibodies against EBV lytic gene products was observed and defined as EBV reactivation *in vivo* [10]. It was also found that serum IgA antibody against EBV is an outstanding feature of NPC [11]. Furthermore, EBV DNA was detected in NPC tissues [12] and various EBV lytic gene products were expressed [13–17]. These findings support the close association of EBV and NPC. Previous works on NPC carcinogenesis have largely been focused on the contributions of EBV latent antigens. Through years of extensive studies, it was concluded that latent EBV participates in the carcinogenesis of NPC after high grade pre-invasive lesion. However, lytic genes have long been suspected also to be involved [18], and the impact of lytic genes on the carcinogenesis of NPC still remains to be elucidated.

Genomic instability (GI) has been defined as a hallmark of cancer and likely contributes to the development of other markers [19]. Previously, using an EBV(+) cell line derived from an NPC patient, which may represent residual NPC cells after remission, we demonstrated that latent EBV infection only induces little GI in the cultured cells and tumorigenesis in non-obese diabetic/ severe combined immunodeficiency (NOD/SCID) mouse after latent passage for 15 cycles. However, after EBV reactivation by TPA/sodium butyrate for 15 cycles, the GI in the cells prominently increased and tumorigenesis in NOD/SCID mouse was profoundly enhanced [20]. We then sought any lytic EBV genes that may contribute to the generation of GI and enhancement of tumorigenesis. We found that the early genes DNase and BALF3 are able to induce GI and progressive tumorigenesis in NPC cells [21, 22]. However, EBV IE genes have not been given attention. The BRLF1 gene is expressed as a 4.0-kb mRNA within 2 hr after viral reactivation, and translated as a 605-amino acid protein [23]. The BRLF1 protein contains an N-terminus region of overlapping DNA binding and dimerization domain and C-terminus of transcription activation domain [24]. BRLF1 activates the transcription of viral genes by directly binding to a GC-rich motif known as the Rta-responsive element (RRE) or indirectly stimulating cell-signaling pathways including phosphatidylinositol 3-kinase (PI3-K) [25], p38 and JNK kinase [26]. To enhance the efficiency of virus replication, many viruses were demonstrated to manipulate the host cell environment, in particular cell cycle progression. Therefore, previous studies focused on how EBV IE gene transcriptions regulate the host cell environment. It was reported that the EBV lytic protein BZLF1 arrested cells in G0/G1 [27], G1/S [28] and G2/M [29]. It has been reported that BRLF1-expressing cells reenters S phase [30]. Our previous studies demonstrated that BRLF1 has ability to interfere with cells at the G1/S transition and induces a cellular senescence [31, 32]. However, there is no study yet to investigate the regulation of BRLF1 in G2 and mitosis phase. Mitosis is a process in cell division and produces copies of genome of daughter cells. The improper distribution of chromosomes during mitosis contributes to GI and malignant transformation of cells [33, 34]. In this study, we used a human nasopharyngeal carcinoma cell line, TW01 cells, derived from the tumor of a Taiwanese patient. TW01 cells may stand for residual NPC cells in patients after remission. We present evidence that the EBV immediate early gene BRLF1 has strong ability to induce genomic instability (GI) by interfering with chromosome segregation and subsequently enhances the tumorigenesis of NPC cells.

## RESULTS

### EBV BRLF1 induces chromosome mis-segregation in NPC cells

**Figure 1:**
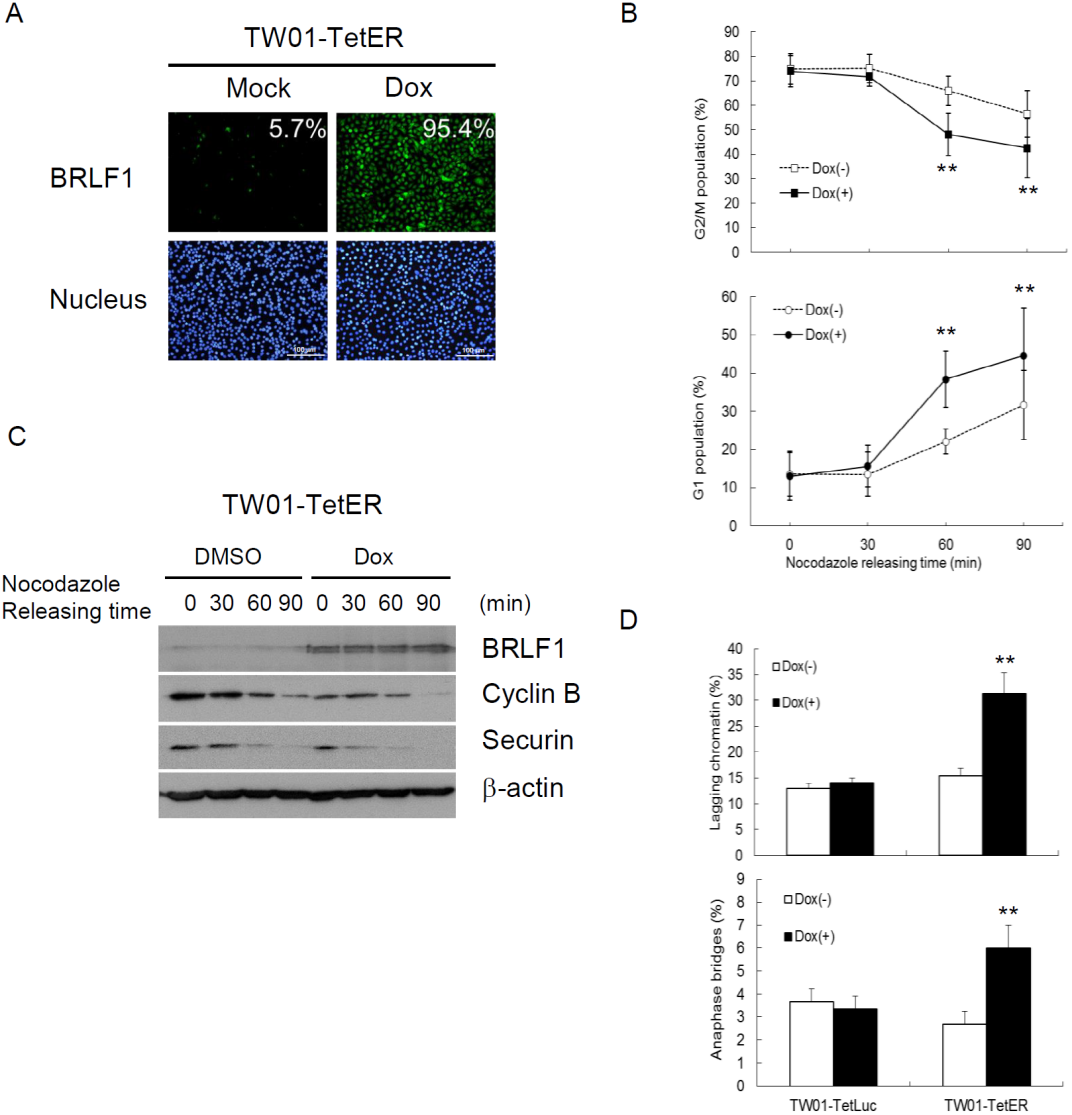
BRLF1 accelerates the process of mitosis in NPC cells **(A)** TW01-TetER cells were treated with 50 ng/ml Dox for 24 h and subjected to immunofluorescence staining. **(B)** TW01-TetER cells were treated with 50 ng/ml Dox and 50 ng/ml nocodazole for 24 h. Nocodazole-arrested TW01-TetER cells were collected by shake-off and released into fresh medium. The samples were collected at the indicated times and cell cycle progression from M to G1 phase was analyzed by flow cytometry. Data are presented as means ± SD. **, *P* < 0.01, compared to Dox(-) treatment at the same time point. **(C)** Levels of BRLF1, cyclin B, securin and β-actin were determined by western blot analysis. **(D)** The samplesat 60 min after nocodazole release were analyzed to determine chromosome-segregation defects. Data are presented as means ± SD.**, *P* < 0.01, compared to Dox(-) treatment of the same cell.

**Figure 2:**
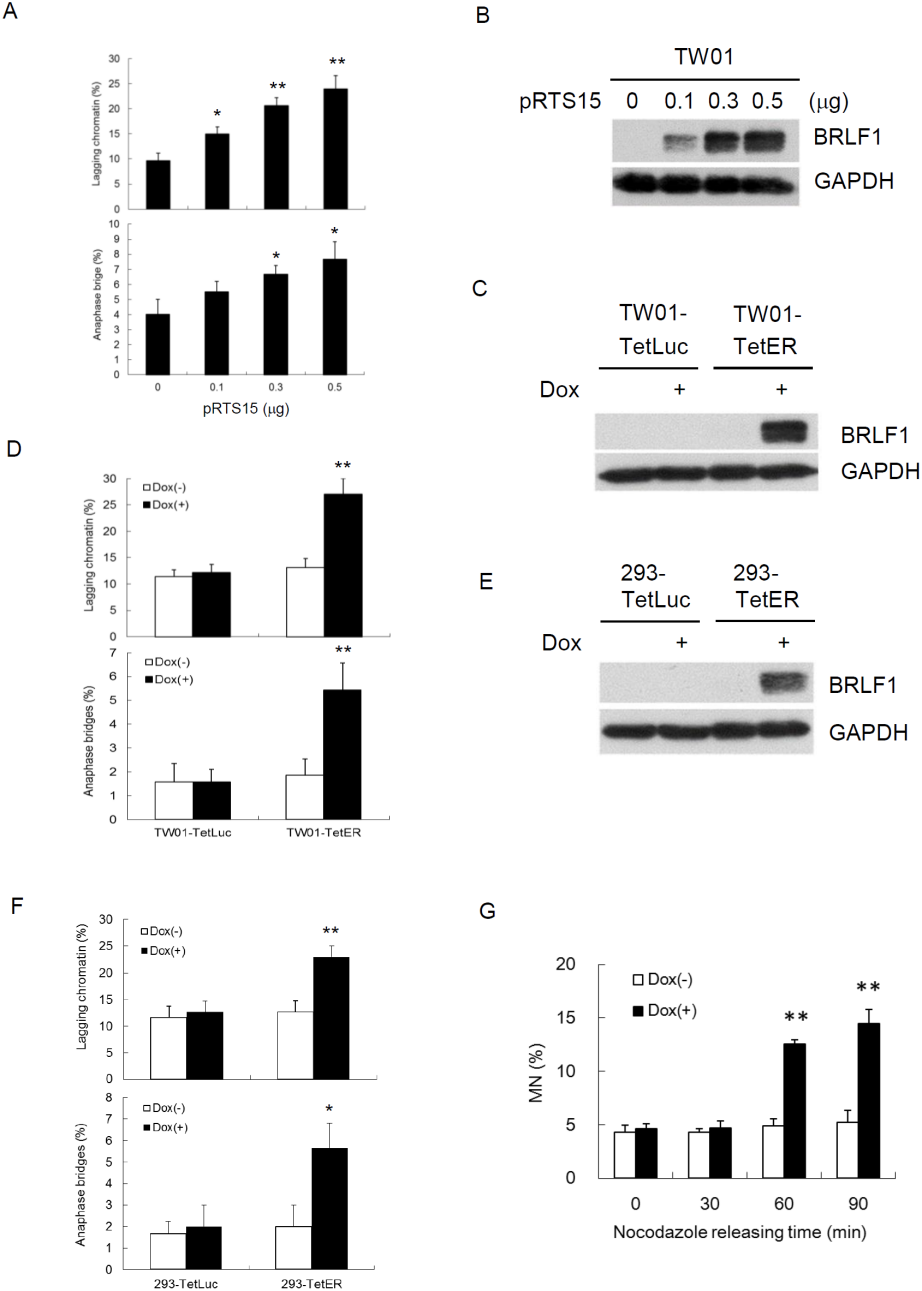
BRLF1 induces chromosome mis-segregation in NPC cells **(A)** TW01 cells were transiently transfected with the doses of pRTS15 for 24 h and chromosome-segregation defects were determined. Data are presented as means ± SD. *, *P* < 0.05; **, *P* < 0.01, compared to mock treatment. **(B)** Cell lysates from (A) were subjected to western blotting analysis. **(C)** TW01-TetLuc and TW01-TetER cells were treated with 50 ng/ml Dox for 24 h and subjected to western blotting, and **(D)** analyzed to determine chromosome-segregation defects. Data are presented as means ± SD. **, *P* < 0.01, compared to Dox(-) treatment of the same cell. **(E)** 293-TetLuc and 293-TetER cells were treated with 50 ng/ml Dox for 24 h and subjected to western blotting, and **(F)** analyzed to determine chromosome-segregation defects. Data are presented as means ± SD. **, *P* < 0.01, compared to Dox(-) treatment of the same cell. **(G)** TW01-TetER cells were treated with 50 ng/ml Dox and 50 ng/ml nocodazole for 24 h. Nocodazole-arrested TW01-TetER cells were collected by shake-off and released into fresh medium. The samples were collected at the indicated times and subjected to micronucleus formation assay. Data are presented as means ± SD. **, *P* < 0.01, compared to Dox(-) treatment at the same time point.

It was revealed that BRLF1 plays an active role in interfering with cell cycle at G0/G1 and S-phases [31, 32]. However, we know very little about the regulation of BRLF1 in mitosis. Because the efficiency of transient transfection with the plasmid is limited, a doxycycline (Dox)-inducible BRLF1 stable clone, TW01-TetER, and a Dox-inducible luciferase stable clone, TW01-TetLuc as control, were established for this experiments. TW01-TetER cells were treated with 50 ng/ml Dox for 24 h and subjected to immunofluorescence staining with BRLF1 antibody (Figure 1A). As shown in Figure 1A, more than 95% of TW01-TetER cells were induced to express BRLF1 under Dox treatment. To determine whether BRLF1 interferes with the process of mitosis, TW01-TetER cells were treated with 50 ng/ml Dox and enriched in mitosis by 50 ng/ml nocodazole treatment for 24 h. The cells were collected by mechanical shake-off and then released to monitor the cell cycle transition from M to G1 phase by flow cytometry. As shown in Figure 1B, the percentage of cells in G2/M phase was 74.8% and 73.9% in mock and BRLF1 expressed cells, respectively. After 60 mins of release from nocodazole, the percentage decreased significantly decreased to 48.1% in BRLF1 expressing cells compared to mock (65.9%). Meanwhile, a prominent enrichment of the G1 phase population was observed in BRLF1 expressing cells (38.4%) compared to mock (22.0%). Clearly, BRLF1 expressing cells efficiently underwent mitotic exit following release from nocodazole, implying that BRLF1 accelerates the process of mitosis in NPC cells. To determine whether BRLF1 is involved in checkpoint inactivation, we examined the degradation kinetics of cyclin B1 and securin in TW01-TetER cells after release from nocodazole. Compared to DMSO treated control cells, cyclin B1 and securin were markedly decreased in BRLF1 expressing cells (Figure 1C), suggesting that BRLF1 interferes with checkpoint inhibition. A defective anaphase checkpoint is well known to increase chromosome mis-segregation. As shown in Figure 1D, after 60 mins of release, the occurrences of chromosome mis-segregation, including lagging chromatin and anaphase bridges, were significantly increased in BRLF1 expressing cells (31.3% and 6.0%) compared to the control (15.3% and 2.7%). These results imply that BRLF1 induces chromosome mis-segregation may be through disturbing and accelerating the process of mitosis. To confirm that BRLF1 induces chromosome mis-segregation, TW01 cells were transiently transfected with various doses of pRTS15 (BRLF1 expression plasmid) and examined for the effect of chromosome mis-segregation. The occasions of lagging chromatin were increased by up to 15, 20.7 and 24% and anaphase bridges increased by up to 5.5, 6.7 and 7.7% (Figure 2A), which correlated with the expression of BRLF1 at doses of 0.1, 0.3 and 0.5 μg, respectively (Figure 2B). The TW01-TetLuc and -TetER cells were treated with 50 ng/ml Dox for 24 h. Then, the cells were subjected to western blot assay staining with BRLF1 antibody (Figure 2C) and also examined for the effect of chromosome mis-segregation. The occurrences of lagging chromatin and anaphase bridge were also significantly increased in BRLF1 expressing TW01-TetER cells compared to the control (27.0% to 13.1% and 5.4% to 1.9%), but were not different in luciferase expressing TW01-TetLuc cells (12.1% to 11.4% and 1.6% to 1.6%, Figure 2D). A similar phenomenon was revealed in BRLF1 expressing 293-TetER cells (Figure 2E and 2F). These results suggest that BRLF1 has the ability to induce chromosome mis-segregation. It is well-known that micronuclei originates predominantly from lagging acentric chromosome or improper segregation of chromatid fragments during mitosis. Interestingly, after 60 mins of release from nocodazole, the number of micronuclei also increased simultaneously with chromosome mis-segregation in BRLF1 expressing TW01-TetER cells (12.6%), compared to the control (4.9%, Figure 2G). These results suggest that BRLF1 induce chromosome mis-segregation may cause a subsequent increase of micronucleus formation.

### EBV BRLF1 induces genomic instability

**Figure 3:**
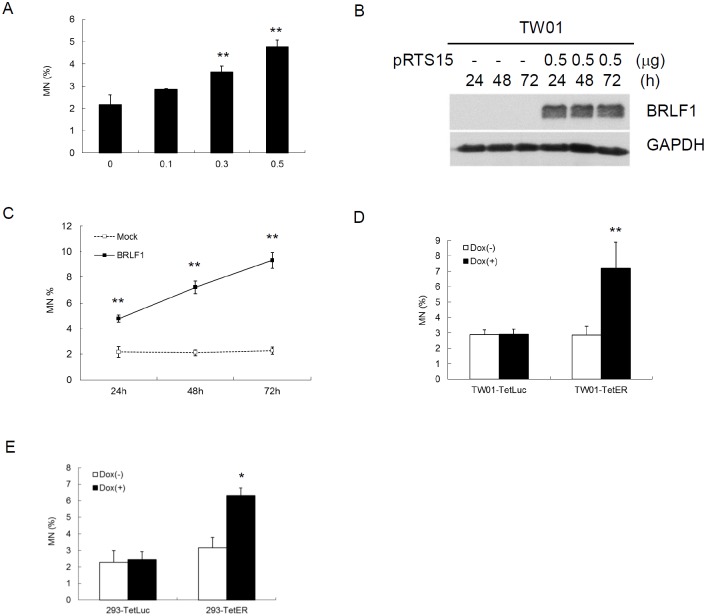
The EBV immediate early gene BRLF1 induces genomic instability **(A)** TW01 cells were transiently transfected with the doses (μg) of pRTS15 for 24 h and micronucleus formation assay was performed. Data are presented as means ± SD. **, *P* < 0.01, compared to mock. **(B)** TW01 cells were transiently transfected with pRTS15 for 24, 48 and 72 h. Western blotting and **(C)** micronucleus formation assay were performed. Data are presented as means ± SD. **, *P* < 0.01, compared to mock treatment at the same time point. **(D)** TW01-TetER cells were treated with 50 ng/ml Dox for 24 h andmicronucleus formation assay was performed. Data are presented as means ± SD. **, *P* < 0.01, compared to TW01-TetER mock treatment. **(E)** 293-TetER cells were treated with 50 ng/ml Dox for 24 h andmicronucleus formation assay was performed. Data are presented as means ± SD. *, *P* < 0.05, compared to 293-TetER mock treatment.

Micronuclei (MN) is a biomarker of genotoxic event and chromosomal instability [35]. To determine whether BRLF1 can induce GI in host cells, TW01 cells were transiently transfected with various doses of pRTS15 and examined for the formation of micronuclei. The numbers of micronuclei were increased by up to 2.9, 3.6 and 4.8% (Figure 3A), correlated with the expression of BRLF1 at doses of 0.1, 0.3 and 0.5 μg, respectively. As cells were transiently transfected with 0.5 μg pRTS15 for 24, 48 and 72 h (Figure 3B), the numbers of micronuclei were increased continually by up to 4.8, 7.2 and 9.3% (Figure 3C). These results suggest that BRLF1 has the ability to induce GI in dose and time-dependent manners. In addition, after TW01-TetER and -TetLuc were treated with 50 ng/ml Dox for 24 h, the numbers of micronuclei were increased significantly in BRLF1 expressing TW01-TetER cells, compared to the control (7.2% to 2.9%), but were not different in luciferase expressing TW01-TetLuc cells (2.9% to 2.9%, Figure 3D). A similar phenomenon was also revealed in BRLF1 expressing 293-TetER cells, (Figure 3E). These results indicate that BRLF1 has the ability to increase GI in cells.

### BRLF1 accelerates the process of mitosis in live zebrafish embryos

**Figure 4:**
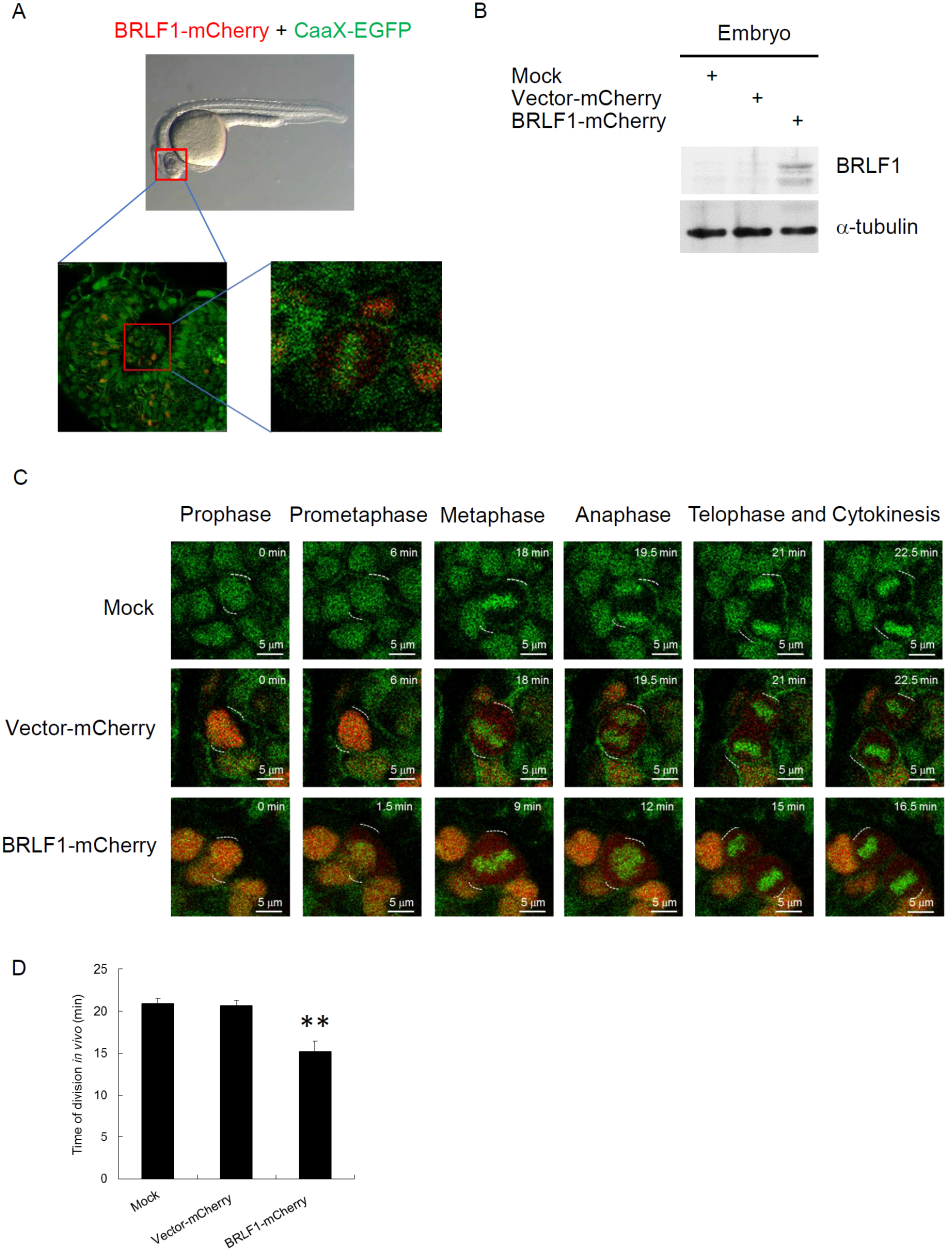
BRLF1 accelerates the process of mitosis in live zebrafish embryos **(A)** Schematic of embryo confocal imaging. **(B)** Western blot analysis for BRLF1 protein levels from wild-type, injected with mRNA encoding vector-mCherry or mCherry-BRLF1 embryo after 24 h injection. **(C)** Time-lapse images were extracted from videos of the uninjected, injected with mRNA encoding vector-mCherry or mCherry-BRLF1 embryos. **(D)** Division time of the uninjected, injected with mRNA encoding vector-mCherry or mCherry-BRLF1 embryo was calculated from nuclear envelope breakdown to the formation of two daughter cells in minutes. Data are presented as means ± SD. **, *P* < 0.01, compared to mock.

To obtain a dynamic analysis under physiological conditions, we monitored chromosome segregation within live zebrafish embryos. Tg (h2afva:h2afva-GFP) embryos at the one-cell stage were injected with mRNA encoding BRLF1-mCherry or vector-mCherry (as a control). After 24 h, the embryos were embedded in low-melt agarose in a coverslip-bottom dish and observed the thinner eye region using time-lapse confocal microscopy (Figure 4A). For detection of BRLF1 expression, the lysates from the embryos were examined by western blotting (Figure 4B). The result showed that BRLF1 protein was successfully expressed in embryos injected with mRNA encoding BRLF1-mCherry. The images extracted from time-lapse videos of vector-mCherry or BRLF1-mCherry expressing embryos are shown in Figure 4C. The result demonstrated that uninjected Tg (h2afva:h2afva-GFP) and vector-mCherry injected embryos undergo normal progression through mitosis. The average division time of both was about 21 mins. However, the cells from BRLF1-mCherry expressing embryos underwent rapid progression (Figure 4C) and the average division time was about 15 mins (Figure 4D). The shortest time of mitosis was even 12 min. This result confirmed that BRLF1 can accelerate the process of mitosis.

### BRLF1 induces GI by chromosome mis-segregation through activation of Erk signaling

**Figure 5:**
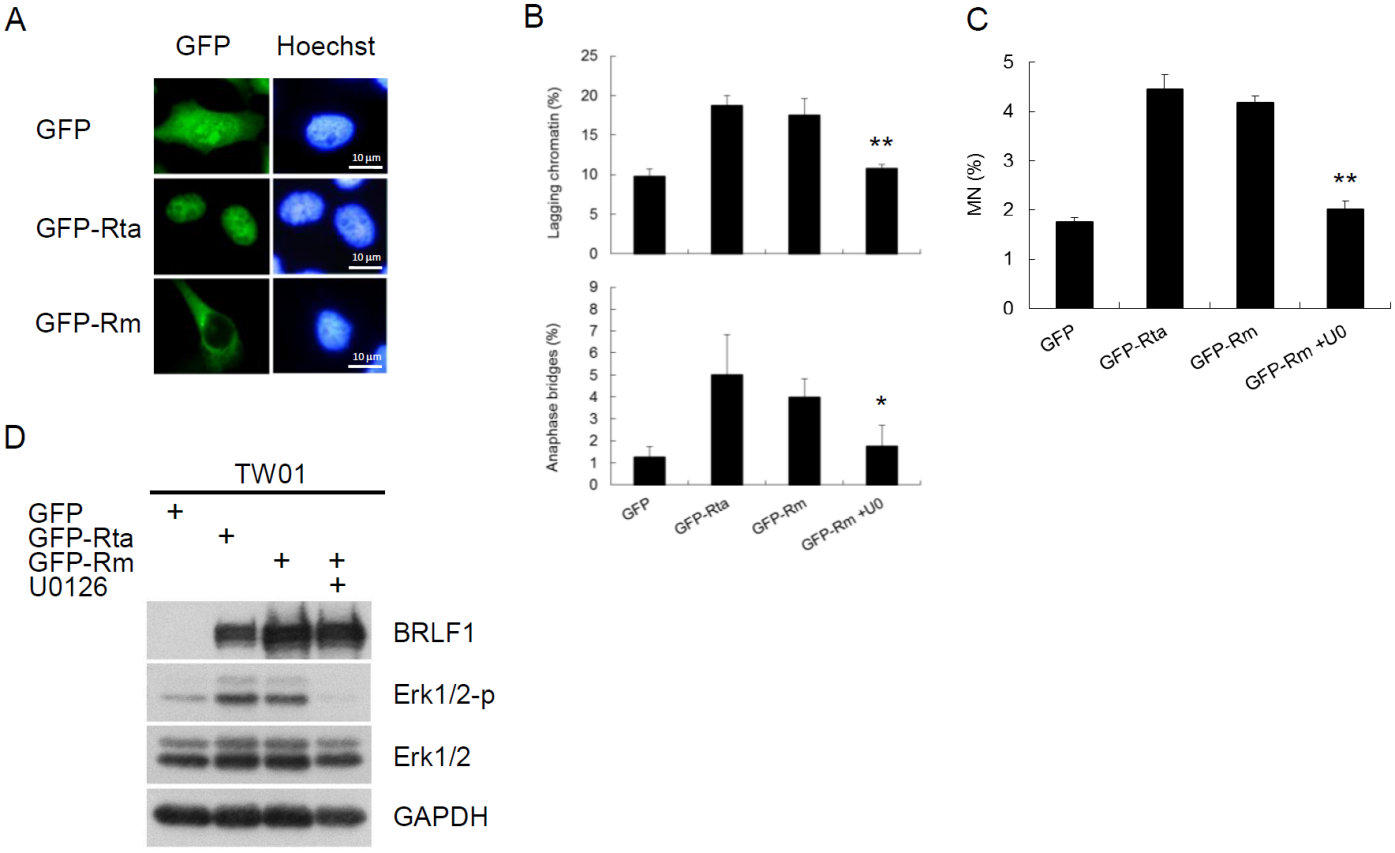
BRLF1 induces chromosome mis-segregation and micronucleus formation by triggering signaling pathway **(A)** Subcellular localization of GFP fusion proteins in TW01 cells. Plasmids were transfected into TW01 cells for 24 h and the cells were examined by fluorescence microscopy. **(B)** TW01 cells were transfected with plasmids and treated with DMSO or 20 μM U0126 for 24 h, then analyzed to determine chromosome-segregation defects and **(C)** micronucleus formation assay.Data are presented as means ± SD. *, *P* < 0.05; **, *P* < 0.01, compared to GFP-Rm transfectant. **(D)** The cell lysatesfrom (B) were subjected to western blot analysis for detection of the indicated proteins.

**Figure 6:**
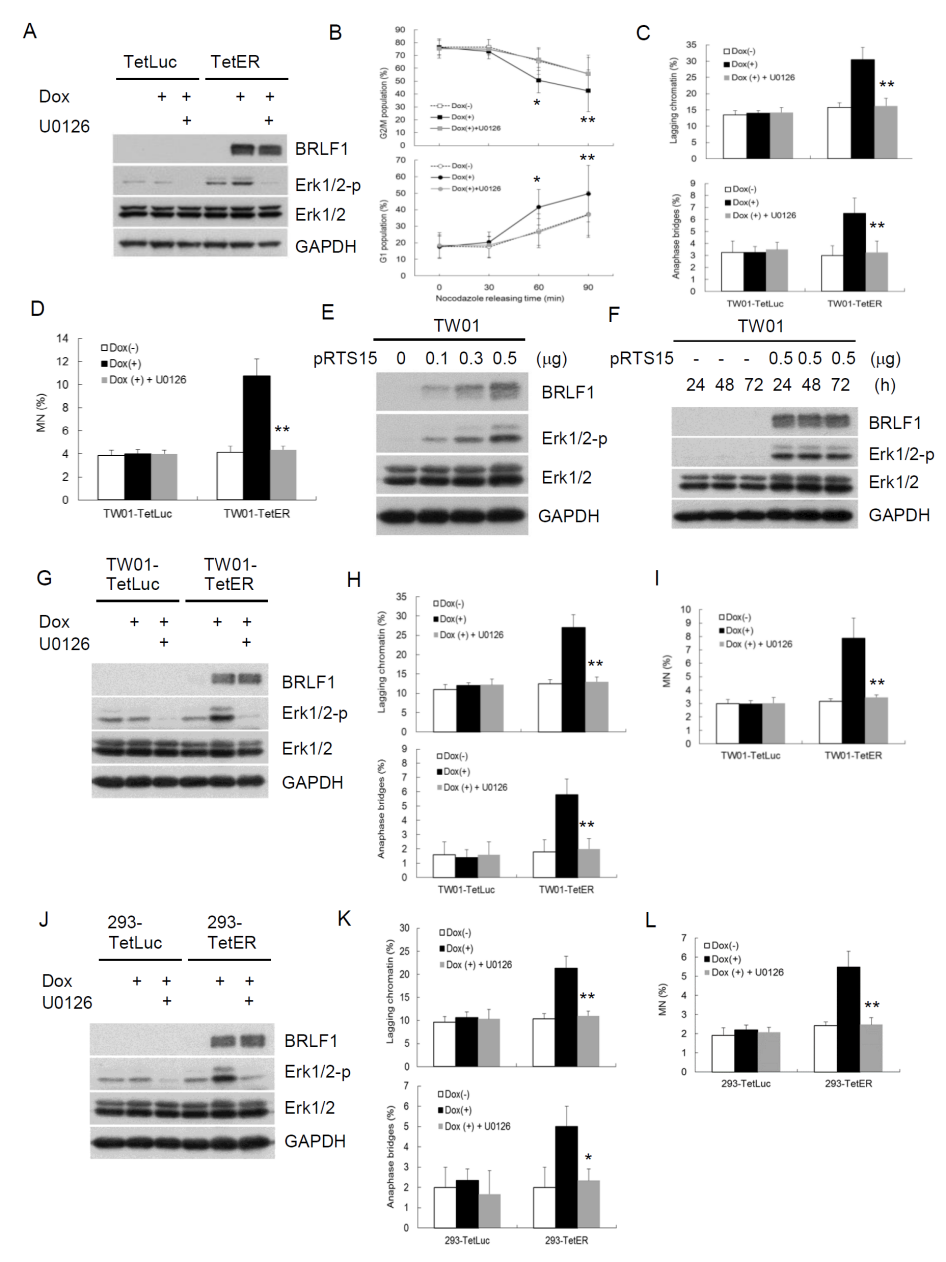
BRLF1 induces GI by chromosome mis-segregation through activation of Erk signaling **(A)** Nocodazole-arrested cells co-treated with 50 ng/ml Dox and 20 μM U0126 for 24 h were collected by shake-off. Western blot analysis was performed to detect the indicated proteins. **(B)** TW01-TetER cells from (A) were then released into fresh medium. The samples were collected at the indicated time and cell cycle profiles were analyzed by flow cytometry. Data are presented as means ± SD. *, *P* < 0.05; **, *P* < 0.01, compared to U0126 treatment at the same time point. **(C)** The samplesat 60 min after nocodazole release were analyzed to determine chromosome-segregation defects and subjected to **(D)** micronucleus formation assay. Data are presented as means ± SD. **, *P* < 0.01, compared to Dox(+) treatment at the same cell. **(E)** TW01 cells were transiently transfected with the doses of pRTS15 for 24 h or **(F)** with 0.5 μg pRTS15 for 24, 48 and 72h. Western blotting was performed to detect the indicated protein levels. **(G)** The cells were co-treated with 50 ng/ml Dox and 20 μM U0126 for 24 h and subjected towestern blotting todetect the indicated protein levels. **(H)** The samples from (G) were analyzed to determine chromosome-segregation defects and subjected to **(I)** micronucleus formation assay. Data are presented as means ± SD. **, *P* < 0.01, compared to Dox(+) treatment at the same cell. **(J)** The cells were co-treated with 50 ng/ml Dox and 20 μM U0126 for 24 h and subjected towestern blotting todetect the indicated protein levels. **(K)** The samples from (J) were analyzed to determine chromosome-segregation defects and subjected to **(L)** micronucleus formation assay. Data are presented as means ± SD. *, *P* < 0.05; **, *P* < 0.01, compared to Dox(+) treatment at the same cell.

BRLF1 is a transcriptional activator. However, BRLF1-mediated transactivation relies on not only direct binding but also the triggering of cellular signaling pathways. To elucidate which mechanism by BRLF1 to induce chromosome mis-segregation and GI, a GFP-Rta nuclear localization signal (NLS) mutant (GFP-Rm, GFP fusion with BRLF1 NLS mutant) was used in this study. GFP-Rta NLS mutant has lost the capacity for nuclear localization, but retains its transactivation ability. The plasmids were transfected into TW01 cells and the cells were examined by fluorescence microscopy after 24 h. GFP-Rta (GFP fusion with BRLF1) located in the nucleus, but GFP-Rm located in the cytoplasm (Figure 5A). However, the increase of lagging chromatin and anaphase bridges was still observed in GFP-Rm expressing cells, compared to the GFP control (17.5% to 9.8% and 4.0% to 1.3%, Figure 5B). The numbers of micronuclei were also significantly increased in GFP-Rm expressing cells (4.2%), compared to the GFP control (1.8%, Figure 5C). Interestingly, the increase of chromosome mis-segregation and micronucleus formation in GFP-Rm expressing cells was almost comparable to GFP-Rta expressing cells, suggesting that BRLF1 induces chromosome mis-segregation and micronuclei by triggering signaling pathways. Several signaling pathways have been reported to be activated by BRLF1. Therefore, we used the inhibitors of these pathways to test which one involving in the induction of chromosome mis-segregation by BRLF1. Interestingly, only the Erk inhibitor, U0126, effectively prevented the effect of BRLF1 (Supplementary Figure 1). As shown in Figure 5D, GFP-Rm induced Erk phosphorylation. However, as U0126 (20 μM) inhibited the phosphorylation (Figure 5D), the induction of chromosome mis-segregation and micronucleus formation was prevented (Figure 5B and 5C). BRLF1 also induced Erk phosphorylation in TW01-TetER cells (Figure 6A). U0126 (20 μM) effectively inhibited the phosphorylation and prevented the acceleration of mitosis from nocodazole release (Figure 6A and 6B). In addition, chromosome mis-segregation (lagging chromatin and anaphase bridges from 30.5% to 16.3% and 6.5% to 3.3%) and micronucleus formation (10.8% to 4.3%) were significantly reduced after 60 mins of release from nocodazole (Figure 6C and 6D). Figure 6E and 6F confirmed BRLF1 significantly induced Erk phosphorylation in a dose- and time-dependent manner in TW01 cells. Erk phosphorylation was also observed in BRLF1 expressing TW01-TetER cells (Figure 6G). U0126 (20 μM) blocked the increase of lagging chromatin and anaphase bridges from 27.0% to 13.0% and 5.8 % to 2.0% (Figure 6H), and the numbers of micronuclei were significantly reduced from 7.9% to 3.5% (Figure 6I). A similar phenomenon was also revealed in BRLF1 expressing 293-TetER cells, (Figure 3J–3L). These results suggest that BRLF1 induces chromosome mis-segregation and GI through activation of Erk signaling.

### Accumulation of GI after recurrent expression of BRLF1

**Figure 7:**
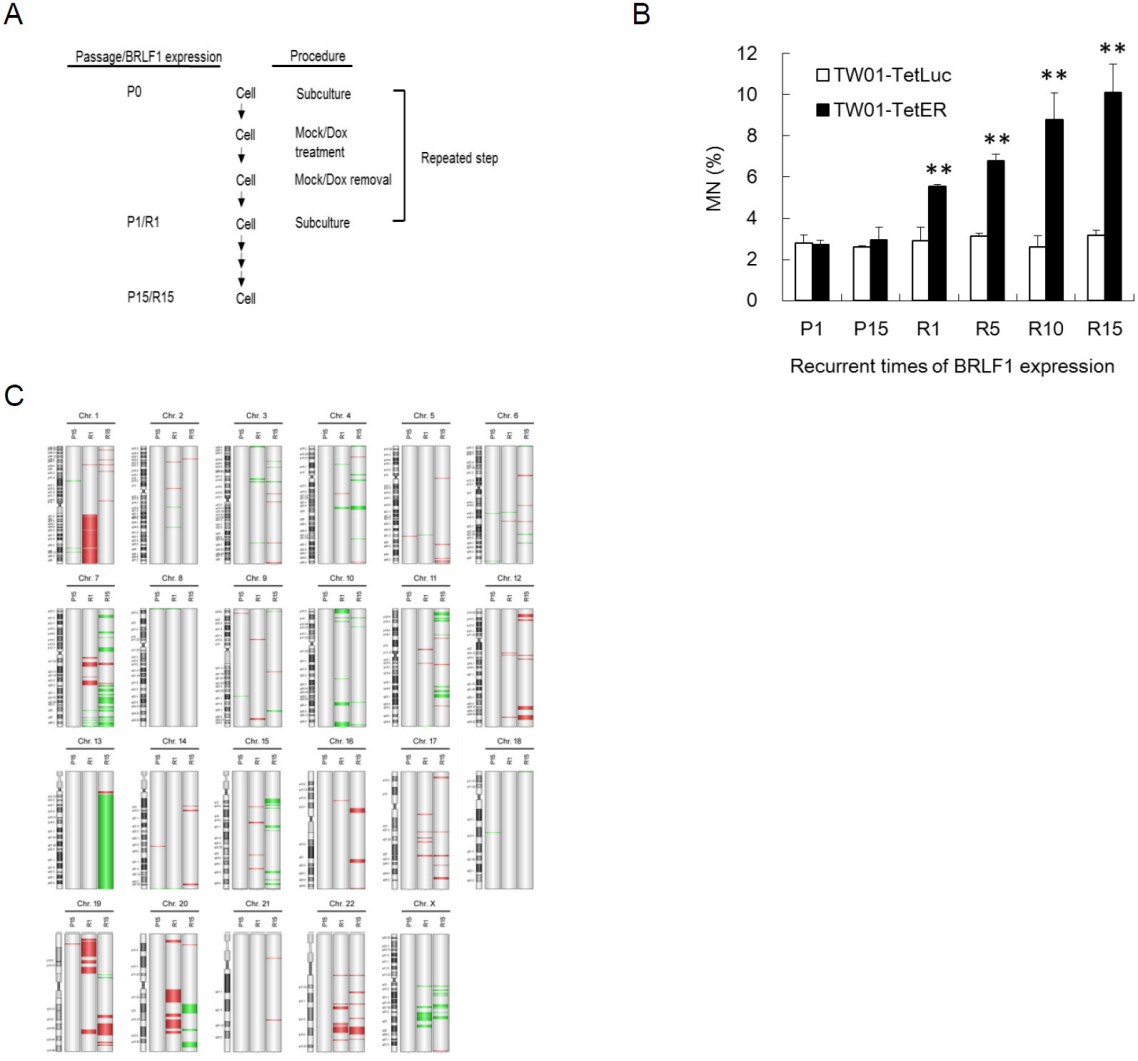
Recurrent BRLF1 expression leads to significant accumulation of genomic instability **(A)** Representative illustration of recurrent EBV BRLF1 expression in NPC cells. **(B)** The cells were harvested at passages 1, 5, 10 and 15 and subjected to micronucleus formation assay. Data are presented as means ± SD. *P* < 0.01, compared to TW01-TetER P1. **(C)** The genomic DNA of cells with mock or Dox treatment at passages 1 and 15 was extracted and subjected to aCGH. TW01-TetER (P1) was used as a common reference. The locations of amplifications and deletions are displayed to the right side of chromosomes with cytobands. Red and green colors indicate amplification and deletion, respectively.

To further test the effects of BRLF1, experiments on long-term recurrent BRLF1 expression were performed. A representative illustration of recurrent BRLF1 expression in NPC cells is shown in Figure 7A. The cells at the beginning were defined as passage 0 (P0). After seeding, cells were mock treated or treated with 50 ng/ml Dox for 24 h. After incubation, the cells were recovered by replacement of fresh medium and incubated for 24 h. The resulting cells were defined as passage 1 (P1) or BRLF1 expression 1 (R1). Following this protocol, recurrent BRLF1 expression was carried out over 15 passages and the cells were harvested at passages 1, 5, 10 and 15. The numbers of micronuclei in TW01-TetER cells after BRLF1 expression were increased by up to 5.5, 6.8, 8.8 and 10.1% for passages 1, 5, 10 and 15, respectively (Figure 7B). In contrast, the number of micronuclei exhibited no difference in TW01-TetLuc cells, regardless of 1 or 15 rounds of Dox treatment (2.9% to 3.2%). This result provides further evidence supporting the concept that BRLF1 induces the accumulation of GI in the host cell population and could be the cause of NPC relapse. Furthermore, array CGH analysis was used for the surveillance of genomic copy number aberrations on the host genome as BRLF1 was expressed recurrently. TW01-TetER cells from P1, P15, R1 and R15 were applied to this analysis. Compared to P1 as a common reference, a dramatic increase of aberrations was observed in R15 especially on chromosomes 3, 4, 5, 6, 7, 11, 12, 13, 14, 15, 16, 17 and 21. However, relatively few aberrations were detected in R1. Under long-term cell culture, P15 did not show obvious changes in terms of aberrations (Figure 7C). According to the results of micronucleus formation assay and array CGH analysis, recurrent expression of BRLF1 may be critical to induce aggravated GI in NPC cells.

### Recurrent BRLF1 expression significantly increases the tumorigenic features of NPC cells

**Figure 8:**
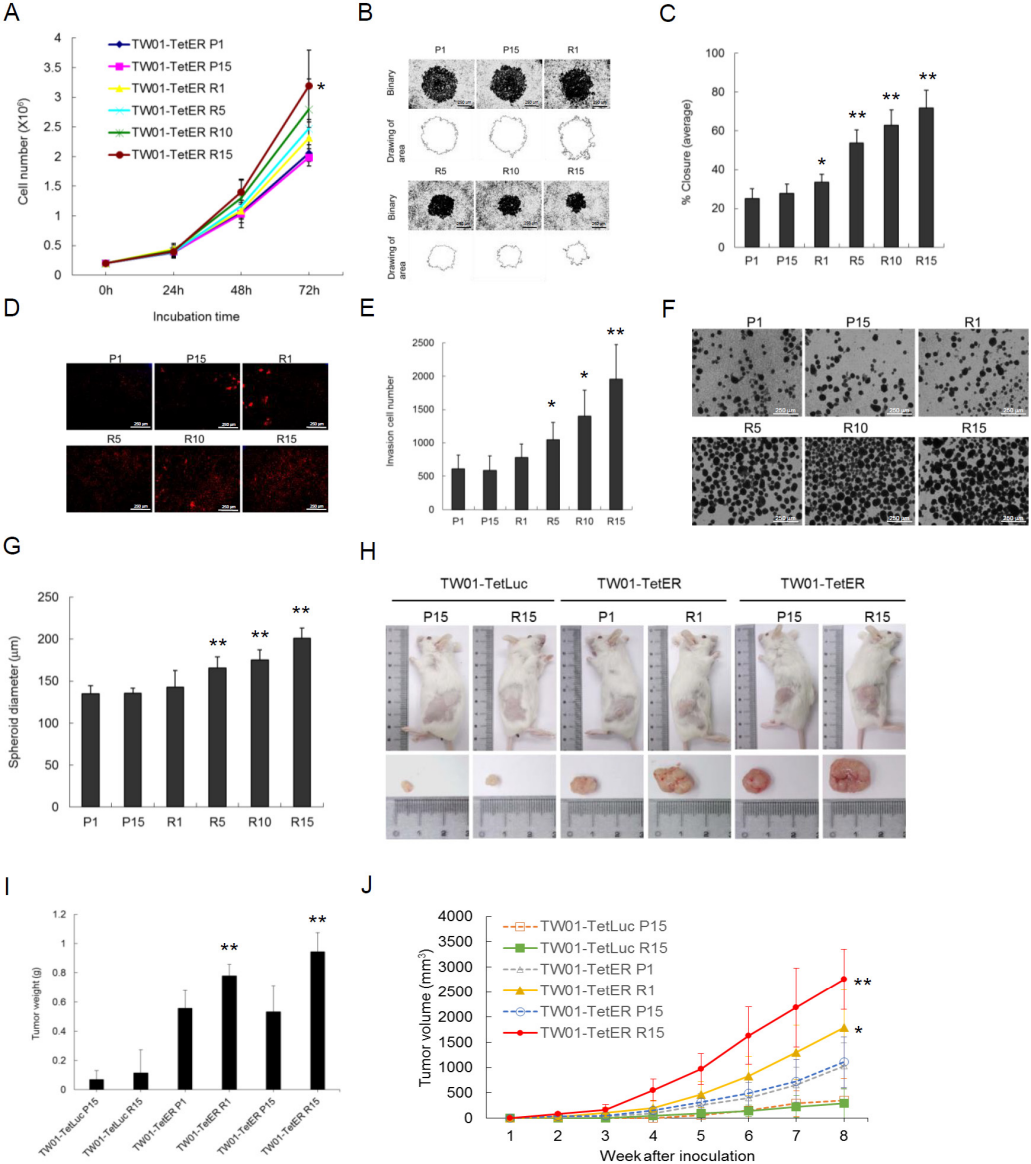
Recurrent BRLF1 expression aggravates the tumorigenic features of NPC cells **(A)** After incubation for 1-3 days, the cells at passages 1, 5, 10 and 15 were harvested and the numbers of viable cells were determined by cell proliferation assay. Data are presented as means ± SD. *, *P* < 0.05, compared to P1. **(B)** The cell migration was determined by the numbers of cells that had migrated into the central blank area after 24 h incubation. The images were captured by microscopy. **(C)** The area of a cell-free zone was measured by digital image processing software ImageJ.Cell migration was determined as percent closure. Data are presented as means ± SD. *P* < 0.05; **, *P* < 0.01, compared to P1. **(D)** The invading cells were visualized using propidium iodide staining and the images were captured by microscopy. **(E)** The numbers of invading cells were counted by ImageJ. Data are presented as means ± SD. *P* < 0.05; **, *P* < 0.01, compared to P1. **(F)** The cells were transferred to 10 cm non-treated plates for 7 days incubation for spheroid assay, and the images were captured by microscopy. **(G)** The diameters of the spheroids were calculated by ImageJ. Data are presented as means ± SD. **, *P* < 0.01, compared to P1. **(H)** The cells harvested at passages 1 and 15 were injected subcutaneously into NOD/SCID mice. Nine animals per group were studied for 8 weeks.Thesacrificed mice and excised tumor nodules were photographed at week 8. **(I)** The excised tumor nodules were weighed. Data are presented as means ± SD. **, *P* < 0.01, compared to TW01-TetER P1. **(J)** The tumor sizes were measured weekly. Data are presented as means ± SD. *, *P* < 0.05; **, *P* < 0.01, compared to TW01-TetER P1.

An increase of genetic changes has been considered to be the cause of carcinogenesis. Aggravated GI may increase the tumorigenic features of cells. Increases of the capacity for proliferation, migration and invasion may contribute to the development of cancers. Because recurrent expression of BRLF1 induced GI in NPC cells, the next important issue is whether this leads NPC cells further toward progressive malignancy. As shown in Figure 8A, a cell proliferation assay was performed. The capability of proliferation from different cells at passages 1, 5, 10 and 15 was determined. As the induction time of BRLF1 was extended, the increased proliferative effect became more prominent. Compared to the P1 cells, a significant increase of proliferation was observed in R15 cells after 72 h incubation, indicating that recurrent BRLF1 expression could increase the proliferation of NPC cells. The cells were also subjected to migration assays. An increase of cell migration following recurrent BRLF1 expression could be seen in Figure 8B. The percentages of closure significantly was increased by up to 33.5, 53.7, 62.8 and 71.7% for R1, 5, 10 and 15, respectively (Figure 8C). Also, an increase of cell invasion could be observed in Figure 8D. The average numbers of invading cells for R1, 5, 10 and 15, were increased to 782, 1045, 1401 and 1956, respectively (Figure 8E). These results reveal that the cells with more rounds of BRLF1 expression exhibit greater migratory and invasive ability, implying that BRLF1 expression is highly correlated with an increase of aggravated properties in the cells. Tumor cells grow as three-dimensional structures in the human body. The formation of multicellular spheroids has been considered to represent the ability of cells to develop into a tumor. Therefore, spheroid formation was used in this study to evaluate the tendency of cells to form tumors. As shown in Figure 8F, the diameter of spheroids of TW01-TetER cells was significantly greater after recurrent BRLF1 expression. The average diameters of the spheroids for R1, 5, 10 and 15, were 143.0, 165.7, 175.5 and 201.2μm, respectively (Figure 8G), suggesting that the potential for intercellular adhesion was also enhanced by recurrent BRLF1 expression.

### Recurrent BRLF1 expression aggravates the tumor progression of NPC cells in NOD/SCID mice

To further evaluate the effect of recurrent BRLF1 expression on tumor growth, a tumorigenicity assay was performed *in vivo* using NOD/SCID mice. The mice were injected with variously treated NPC cells and the tumor volumes were monitored weekly. The tumor nodules were taken from the mice at week 8. The results showed that TW01-TetER cells after BRLF1 expression (R1 and R15) have larger tumor sizes (Figure 8H). The weights of the tumor nodules were significantly increased to 0.75 g and 1.03 g from TW01-TetER cells R1 and R15, compared to 0.56 g and 0.53 g from TW01-TetER cell P1 and P15, respectively (Figure 8I). As shown in Figure 8J, there is no obvious correlation between tumor volumes and passage numbers of the TW01-TetER cells P1/P15 or Dox treatment of TW01-TetLuc cells P15/R15, indicating long-term cultivation or recurrent Dox treatments have no significant effect on the tumor growth of NPC cells. In contrast, steady and significant increases of tumor volumes were observed in the mice bearing tumors from the cells with BRLF1 expression (TW01-TetER R1 and R15 cells). Dramatically increased tumor sizes were observed at week 8 in mice inoculated with TW01-TetER R1 (1789.5 mm^3^) and R15 cells (2754.9 mm^3^), as compared with tumors obtained from mice inoculated with TW01-TetER P1 (1033.5 mm^3^) and P15 cells (1110.3 mm^3^). This indicates that, after BRLF1 expression, the cells acquired the ability to grow more actively *in vivo*. Taken together, these results show that the aggravation of NPC cells is proportional to the rounds of BRLF1 expression and contributes to the progressive malignancy of the tumors.

## DISCUSSION

EBV infection is associated with many human malignancies, including Burkitt’s lymphoma, post-transplant lymphoproliferative disease (PTLD) and nasopharyngeal carcinoma [8]. In Burkitt’s lymphoma, it has been suggested latent EBV infection contributes to the promotion of genomic instability and subsequent carcinogenesis [36, 37]. On the contrary, lytic genes contribute most importantly to the induction of PTLD [38]. It was demonstrated recently that the EBV immediate early gene BZLF1 exerts most important effects in the development of EBV-positive lymphomas in an abortive lytic form [39, 40]. For the contribution of EBV to NPC, it has longtime been believed that latent EBV infection contributes most significantly to the carcinogenesis [18]. In addition to the contributions of EBV latent genes [41–44] to the carcinogenesis of NPC, it has long been suspected that lytic genes also may be involved. Abortive expression of EBV genes was revealed in NPC biopsies and nude mice transplants [45, 46]. It also was concluded that most NPC featured an abortive EBV lytic cycle [14]. More strikingly, the immediate early gene BRLF1 was detected in NPC biopsies by immunohistochemistry staining [47] and RT-PCR [16, 46]. The antibodies against BRLF1 were detected in 83% of NPC plasma samples but only 1.9% in controls [15]. Nowadays, serum antibodies against BRLF1 becomes a novel biomarker for the screening and diagnosis of patients with NPC [48–50]. These data suggested that BRLF1 may be an important factor in the pathogenesis of NPC. BRLF1 was shown to induce reactivation of EBV [51–53] and also was found to cooperatively function with BZLF1 in the transcription of EBV genes [54, 55]. However, how BRLF1 contributes to the carcinogenesis has not been elucidated yet. Our previous studies demonstrated that ectopic overexpression of BRLF1 arrests cells at the G1/S transition and elicits a cellular senescence program [31, 32]. However, rapidly growing small cells devoid of SA-β-Gal expression were found after a further long culture [32]. As shown in Supplementary Figure 2, we confirmed that BRLF1 expression for 8 days induces a significant cellular senescence (45.9% or 70.6% in TW01-TetER or 293-TetER cells, respectively). However, EBV lytic genes are expressed in a temporally regulated cascade. Once lytic cycle is activated, BRLF1 expression occurs very rapidly (2 hours or less) and then quickly decreases within 24 hours [56–58]. Therefore, BRLF1 protein should not exist for too long. In this study, expression of BRLF1 for short-term (24h) only induced cellular senescence in a small amount of cells (1.2% or 2.1% in TW01-TetER or 293-TetER cells, respectively). Recurrent expression of BRLF1 for 15 rounds still only led a small amount of cells to the cellular senescence (3.2% or 7.3% in TW01-TetER or 293-TetER cells, respectively). Therefore, we believe that the aggravated NPC cells should come from those cells that were not going cellular senescence. Those cells were still able to repeatedly go into mitosis, and accumulated GI and tumorigenic phenotypes by BRLF1. The NPC cells even could grow faster (Figure 8A). Here, we found that BRLF1 accelerates the process of mitosis and induces genomic instability by interfering chromosome segregation. Chromosome aberrations might cause dysregulation of gene expression and promote cells to progressive malignancy. It will be another important question to explore the changes in gene expression that results from BRLF1 expression.

Defective control of mitosis is a major cause of chromosome mis-segregation and subsequent micronucleus formation [59]. In this study, we demonstrated that BRLF1 expressing cells undergo fast mitotic exit from nocodazole release (Figure 1B). The mitotic checkpoint is regulated by degradation of cyclin B and securin which allows the sister chromatids to separate [60]. As shown in Figure 1C, cyclin B1 and securin were markedly decreased in BRLF1 expressing cells after nocodazole release, suggesting that BRLF1 may compromise the checkpoint signal and induce aberrant mitotic exit. Thus, it may lead to chromosomal mis-segregation and the consequent formation of micronuclei. Indeed, aberrant mitotic exit and a significant increase of chromosome mis-segregation were observed after 60 mins of nocodazole release (Figure 1D). Meanwhile, significant increase of micronucleus formation was also observed at the same time point (Figure 2G). The phenomenon that BRLF1 induced chromosomal mis-segregation (Figure 2D and 2F) and micronucleus formation (Figure 3D and 3E) was also observed in TW01-TetER and 293-TetER. Furthermore, it was in a dose-dependent manner (Figure 2A and 3A). These results indicate that BRLF1 induces and accumulates GI in cells by interfering with the mitotic process to induce chromosome mis-segregation. To corroborate this observation, a study in zebrafish model was carried out. The mCherry-BRLF1 expressing cells in live zebrafish embryos also underwent a rapid progression of mitosis (from 21 to 15 mins), indicating that BRLF1 also induces aberrant mitotic exit under normal physiological conditions (Figure 4). Furthermore, we observed that BRLF1 could significantly override nocodazole and taxol induced mitotic arrest (Supplementary Figure 3), implying that BRLF1 may cause defects in activation of the mitotic checkpoint.

Since we have shown that EBV early genes DNase and BALF3 induce GI [21, 22], we wanted to determine whether IE genes have the same ability. The results showed that BZLF1 does not have that ability, however, BRLF1 induces GI quite strongly. A similar phenomenon was revealed in BRLF1 expressing TW01-TetER and 293-TetER cells (Figure 3), suggesting the effect of BRLF1 is not only specific to NPC cells but also other epithelial cells. It has been suggested the major site of lytic EBV replication in the human host is epithelial cells [61]. BRLF1 was demonstrated to be activated by the transcriptional factor Sp1, but the BRLF1 promoter cannot be actived in B cells [62]. It suggests that host cell factor(s) may be very important for BZLF1 and BRLF1 to exert their biological function.

BRLF1 is a transcription factor, so we expected it would exert the function through regulation of nucleolocalization. However, we found that the GFP-Rm, loses its nuclear localization (Figure 5A), still induces chromosome mis-segregation and micronuclei in cells (Figure 5B and 5C), suggesting BRLF1 may function through other cellular factor(s). Several studies demonstrated that many signaling factors are used by BRLF1 to exert its function. Here, we found that the Erk inhibitor significantly inhibits the acceleration of mitosis (Figure 6B) and increase of chromosome mis-segregation and micronucleus formation (Figure 6C and 6D). The similar inhibitory effects were observed in GFP-Rm expressing TW01 cells (Figure 5B and 5C), BRLF1 expressing TW01-TetER (Figure 6H and 6I) and 293-TetER cells (Figure 6K and 6L). The results suggest that BRLF1 induces chromosome mis-segregation and micronucleus formation by activation of Erk signaling. Although it had been shown that Erk activity is involved in the regulation of mitosis in mammalian cells [63], hyperactivation of Erk had been reported to perturb mitotic progression, leading to abnormal mitotic spindles and chromosomal abnormalities [64]. However, details of the role of Erk in the regulation of mitosis remain unclear. We are currently working to delineate the mechanisms.

In this study, we observed that BRLF1 induces multiple chromosomal abnormalities (Figures 1-3). The results of aCGH analysis also showed the regional copy-number alterations are increased progressively in direct proportion to the rounds of BRLF1 expression, specifically at chromosome 7, 11, 12 and 13 (Figure 7C). The cells had progressive tumorigenic features, including increase of proliferation, cell migration, invasion, formation of spheroids and development into larger tumor nodules in NOD/SCID mice (Figure 8). BRLF1 is one of the first viral proteins expressed during EBV reactivation. So it is possible that, in the early stage of EBV reactivation in residual NPC cells, BRLF1 causes mis-segregation of chromosomes in mitosis. Then, recurrent BRLF1 expression may aggravate GI to increase the tumorigenic features of host cells and contribute to the development of tumors.

Taken together, in this study we demonstrate that the EBV IE gene, BRLF1, is able to induce GI and accumulate tumorigenic phenotypes of NPC cells by interfering with chromosome segregation. It may be the important factor contributing to initiation of relapse and metastasis of NPC. Therefore, BRLF1 may be a unique target for prevention as well as retardation of relapse of NPC after remission.

## MATERIALS AND METHODS

### Cell lines

TW01 (From Dr. C. T., Lin’s lab, Taiwan University and Hospital, Taiwan) is a human nasopharyngeal carcinoma cell line, which has lost the EBV genome [65]. TW01-TetLuc and TW01-TetER (From Dr. S. F. Lin’s lab, National Health Research Institutes, Taiwan) are doxycycline inducible luciferase and EBV BRLF1 conditional expression cell lines, respectively. Both cell lines were established from TW01-Tet cells, which express stably the tetracycline repressor, by respectively transfected with pLenti4 (Invitrogen) or pLenti4-BRLF1, and selected with 25 μg/ml blasticidin (Sigma-Aldrich) and 500 μg/ml zeocin (Invitrogen). Same procedures were carried out to establish 293-TetLuc and 293-TetER from 293 cells (From Dr. S. F. Lin’s lab, National Health Research Institutes, Taiwan).

### Detection of genomic instability

Micronucleus formation was evaluated as described previously [20]. Cells seeded onto coverslips were fixed with 100% methanol and DNA was stained with 1 μg/ml Hoechst 33258 (Sigma-Aldrich) for 5 min. Micronuclei, chromosomal laggings and mitotic bridges in cells were examined using a fluorescence microscope (Olympus). At least 1,000 cells were counted for the evaluation of micronucleus occurrence and at least 100 mitotic cells were counted for evaluation of chromosomal laggings and mitotic bridges in each sample.

### Western blot analysis

Cells were lysed in lysis buffer containing 3.3% SDS, 1.67M urea and 4.4% 2-β-mercaptoethanol and then running onto 10% SDS-polyacrylamide gels. The protein bands were electrophoretically transferred to Hybond-C super membranes (Amersham), and probed with primary antibodies and followed with a horseradish peroxidase-conjugated secondary antibody. Finally, the signals were detected using an enhanced chemiluminescence substrate (PerkinElmer) and exposure to X-ray film (Fujifilm). Anti-BRLF1 antibody was obtained from Argene. Anti-phospho-Erk1/2 (Thr202/Tyr201), anti-Erk1/2 and anti-β-actin antibodies were purchased from Cell Signaling Technology. Anti-cyclin B antibody was obtained from Santa Cruz. Anti-securin and anti-GAPDH antibodies were obtained respectively from Abcam and GeneTex.

### Flow cytometry analysis

Aliquots of cells (1x10^6^ /ml) were washed with PBS and fixed with 75% ethanol for at least 2 h at -20°C. The fixed cells were repelleted by centrifugation and permeabilized in 1 ml of 0.2% Triton X-100/PBS solution for 10 min. After centrifugation, the cells were resuspended in PBS containing 10 μg/ml RNase A and 10 μg/ml propidium iodide for 30 min incubation. The analysis was performed using a FACScan flow cytometer (FACScan; Becton Dickinson). At least ten thousand events were collected from each sample and analyzed using the CellQuest software.

### Microinjection into zebrafish embryos

Oligonucleotide primers (Forward: 5’-CGAAGATCTCTAATACGACTCACTATAGGGC-3’, Reverse: 5’-AGCAAGTTAAATAAGCTGGTGTCAAAAATAGAC-3’) were synthesized and used to amplify BRLF1 gene by RT-PCR. The BRLF1 cDNAs were purified using a QIAquick PCR Purification Kit (Qiagen), cleaved with the restriction endonucleases (BglII and HindIII), and cloned into pmCherry-N1 Vector (Clontech). The capped sense BRLF1-mCherry and CaaX-EGFP mRNA (to label membrane with EGFP) were respectively transcribed by using the mMessage mMachine T7 kit and SP6 kit (Life Technologies). The synthesized mRNAs with 2.3 nl of 200 ng/μl BRLF1-mcherry and 120 ng/μl Caax-EGFP were dissolved in 0.2% phenol red and then microinjected into Tg (h2afva:h2afva-GFP) embryos (From Taiwan Zebrafish Core Facility at National Health Research Institutes, Zhunan, Taiwan) at one cell stages using an IM 300 Microinjector (Narishige). After 24 h, embryos were anesthetized using 0.4% tricaine and embedded in a 1% low-melt agarose. The live images of cell mitosis in eyes of embryo were visualized by the Leica TCS SP5II AOBS Confocal Microscope and recorded using the digital camera.

### Recurrent expression of EBV BRLF1

The procedure was carried out as described previously [22]. TW01-TetLuc and TW01-TetER cells were cultured in Dulbecco’s modified Eagle’s medium (DMEM) supplemented with 10% tetracycline-free fetal bovine serum (Invitrogen) and incubated at 37°C and 5% CO_2_. To maintain the selected clones, the medium was supplemented with 12.5 μg/ml blasticidin (Sigma-Aldrich) and 250 μg/ml zeocin (Invitrogen). For the induction of luciferase or BRLF1 expression, TW01-TetLuc or TW01-TetER cells were treated with doxycycline (Dox, Sigma-Aldrich). The cells were seeded and incubated in DMEM supplemented with 10% tetracycline-free fetal bovine serum at 37°C and 5% CO_2_ for 24 h, and then mock treated or treated with 50 ng/ml Dox for another 24 h. After incubation, the cells were recovered by replacement of fresh medium and incubated for 24 h. The cells were trypsinized and reseeded for the next cycle. ‘‘Pn’’ represents for mock treated cells, ‘‘Rn’’ represents for Dox treated cells and “n” represents for the passage number of the cells. For example, the TW01-TetER cells from the first cycle were defined as passage 1 (P1) or BRLF1 expression 1 (R1). In this study, the induction was performed up to 15 cycles of expression.

### Cell proliferation assay

Cells were seeded onto 6-well plates. Every 24 h total for 3 days, the cells were harvested and enumerated on a haemocytometer. The number of viable cells was determined on the basis of exclusion by using 0.4% Trypan Blue.

### Cell migration assay

The assay was carried out according to the manufacturer’s instructions (Platypus Technologies). Cells were seeded onto 96-well plates containing Oris stoppers and incubated overnight to confluence at 37°C and 5% CO_2_. The stoppers were removed and the cells were incubated for another 24 h to permit cell migration. The bright-field images were captured using a fluorescence microscope (Olympus). The areas of a cell-free zone were determined using digital image processing software ImageJ (National Institutes of Health). The cell migration was presented as percent closure and calculated using the formula: [(pre-migration) area- (migration) area/ (pre-migration) area] × 100.

### Cell invasiveness assay

The HTS FluoroBlok inserts (Falcon, Cambridge, MA) were first coated with Matrigel (Becton Dickinson, Franklin Lakes, NJ). Cells in 2% FBS-containing DMEM were seeded onto the Matrigel-coated membranes. The inserts were set in 24-well plate with 10% FBS-containing DMEM for 24 h incubation. In turn, the membranes were fixed with 100% methanol and stained with 50 μg/ml propidium iodide (Sigma-Aldrich) at room temperature for 10 min. The cells invaded to the lower surface of the membrane were photographed by a fluorescence microscope. The cell numbers were calculated by ImageJ.

### Spheroid formation assay

The cell suspension with 5 × 10^4^ cells was transferred to 10 cm non-treated plates and incubated at 37°C and 5% CO_2_. Spheroids were collected by brief centrifugation on the 7th day and bright-field images were photographed using a fluorescence microscope (Olympus). The diameters of spheroids were calculated by ImageJ.

### *In vivo* tumorigenesis assay

Six-week-old NOD/SCID female mice (BioLASCO Taiwan Co., Ltd., Taipei, Taiwan) were divided into three groups of nine and 2 × 10^6^ cells suspended in serum-free DMEM were injected subcutaneously into the right or left dorsal flanks of mice. Mice were monitored weekly. The length (l) and width (w) of tumor were measured by calipers. The tumor sizes were estimated using the following formula: tumor volume = l w^2^ × 0.52. Mice were sacrificed as the maximum tumor diameter reaching approximately 20 mm. The tumors were removed and weighed.

### Ethics statement

The protocols of zebrafish (NHRI-IACUC-105026-A) and mice work (NHRI-IACUC-105025-A) were approved by the Institutional Animal Care and Use Committee of National Health Research Institutes (IACUC) at Taiwan and were carried out according to the recommendations in the Guide for the Care and Use of Laboratory Animals of the National Institutes of Health.

### Array-based comparative genomic hybridization analysis (Array CGH)

Genomic DNA was purified from cells using a DNeasy Tissue Kit (Qiagen) and subjected to commercial SurePrint G3 Human CGH Microarray Kit 1×1M (Agilent Technologies). Following steps were performed according to the manufacturer’s instructions. The data extraction was performed by Agilent Genomic Workbench version 7.0.4.0. The aberrant regions were determined using Z-score statistical algorithm with moving an average window of 5 Mb. The Z-score threshold was set at 2.5 to make an amplification or deletion for each altered locus.

### Statistical analysis

Data are presented as means ± standard deviations for at least three independent experiments. Student’s *t* test was used for comparisons of two groups. p < 0.05 was considered to be statistically significant.

## SUPPLEMENTARY MATERIALS FIGURES


