# Supplementary material for: Epstein–Barr virus BRLF1 induces genomic instability and progressive malignancy in nasopharyngeal carcinoma cells
Source: Oncotarget. 2017 Sep 5;8(45):78948–64. doi: 10.18632/oncotarget.20695 (PMC5668011; doi:10.18632/oncotarget.20695)
Supplement: Supplementary file 1 [file oncotarget-08-78948-s001.pdf]

Epstein–Barr virus BRLF1 induces genomic instability and progressive malignancy in nasopharyngeal carcinoma cells

SUPPLEMENTARY MATERIALS

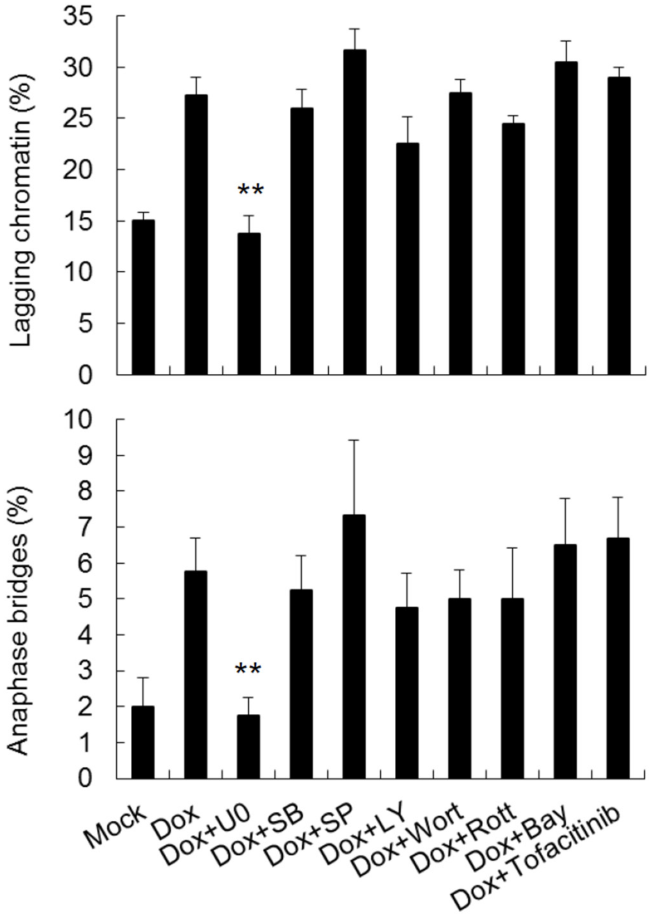

Supplementary Figure 1: Erk inhibitor U0126 effectively prevents chromosome mis-segregation in BRLF1 expressing TW01-TetER cells. TW01-TetER cells were co-treated with 50 ng/ml Dox and various inhibitors, including 20 μM U0126 (ERK inhibitor), 20 μM SB203580 (p38 MAPK inhibitor), 10 μM SP600125 (JNK inhibitor), 10 μM LY294006 (PI3K inhibitor), 1 μM wortmannin (PI3K inhibitor), 5 μM rottlerin (PKC inhibitoror), 10 μM Bay 11-7082 (NFκB inhibitor), 1 μM Tofacitinib citrate (JAK inhibitor) for 24 h and subjected to determine chromosome-segregation defects. Data are presented as means ± SD. **, $P < 0.01$; *, $P < 0.05$, compared to Dox treatment.

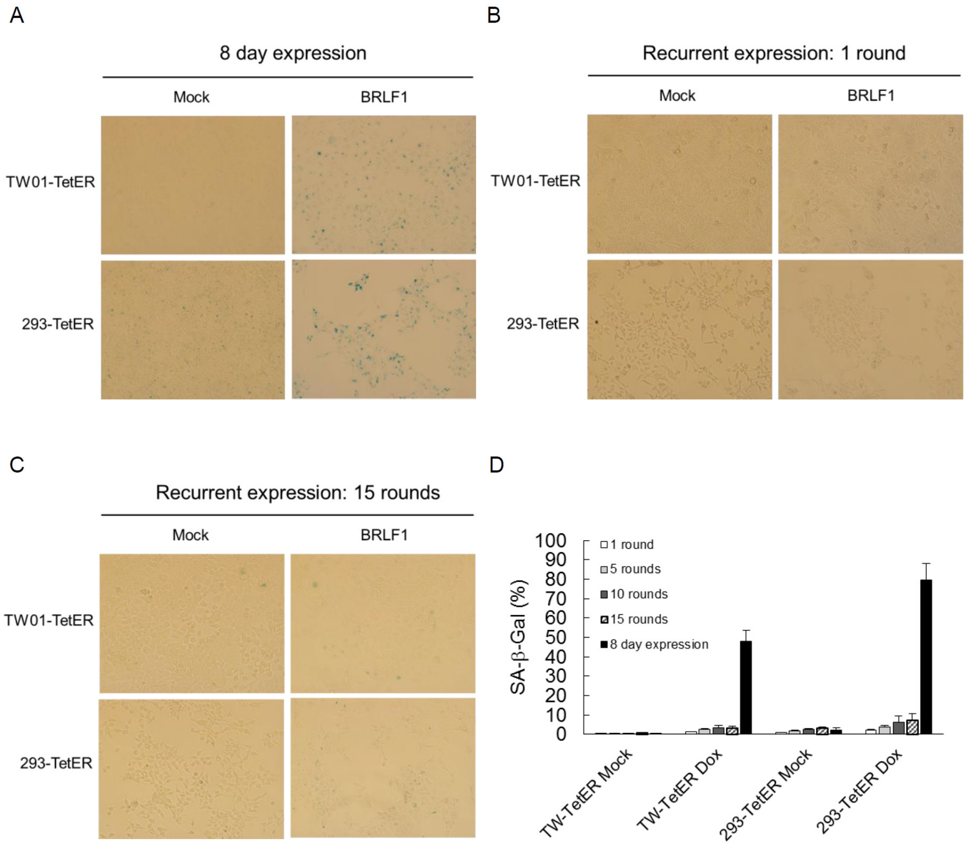

Supplementary Figure 2: Recurrent and short-term expression of BRLF1 only induce cellular senescence in a small amount of cells. Senescence associated β galactosidase (SA-β-Gal) staining were performed. Photography of mock or Dox-treated cells was illuminated at 200X magnifications. **(A)** After seeding, TW01-TetER and 293-TetER cells were mock treated or treated with 50 ng/ml Dox for 8 days and subjected to SA-β-Gal staining. **(B)** After seeding, the cells were mock treated or treated with 50 ng/ml Dox for 24 h. After incubation, the cells were recovered by replacement of fresh medium and incubated for another 24 h. The resulting cells were defined as one round of BRLF1 expression (1 round) and subjected to SA-β-Gal staining. **(C)** Following this protocol, recurrent BRLF1 expressions were carried out over 15 passages (15 rounds) and subjected to SA-β-Gal staining. **(D)** The percentage of SA-β-Gal staining-positive cells was averaged from three randomly selected fields. Data are presented as means ± SD

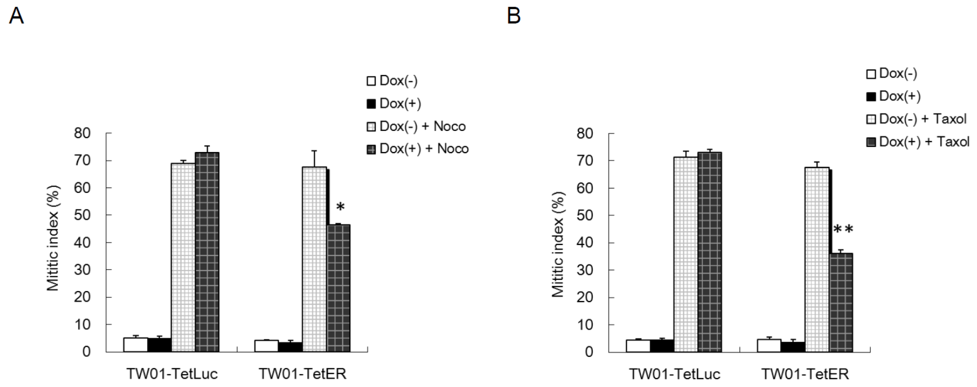

Supplementary Figure 3: BRLF1 expression significantly overrides nocodazole- and taxol-induced mitotic arrest. (A) TW01-TetLuc and TW01-TetER cells were co-treated with 50 ng/ml Dox and 100 ng/ml nocodazole (Noco) or **(B)** 100 nM paclitaxel (Taxol) for 24 h. The cells were harvested, fixed and stained with 1 μg/ml Hoechst 33258. The percentage of mitotic cells in 1000 cells was determined as the mitotic index (%). Data are presented as means ± SD. **, $P < 0.01$; *, $P < 0.05$, compared to Dox(-) + Noco or Taxol treatment at the same cell.
